# Farrerol Enhances Nrf2-Mediated Defense Mechanisms against Hydrogen Peroxide-Induced Oxidative Damage in Human Retinal Pigment Epithelial Cells by Activating Akt and MAPK

**DOI:** 10.1155/2021/8847844

**Published:** 2021-03-03

**Authors:** Ning Ma, Xiaolin Yang, Chong Qi, Qinlei Yu, Chao Zhu, Hua Ren

**Affiliations:** ^1^Department of Ophthalmology, The First Hospital of Jilin University, Changchun 130001, China; ^2^Department of Geriatrics, The First Hospital of Jilin University, Changchun, China; ^3^Institute of Translational Medicine, The First Hospital of Jilin University, Changchun, China; ^4^Jilin Provincial Animal Disease Control Center, 4510 Xi'an Road, Changchun 130062, China; ^5^Department of Ophthalmology, The Second Hospital of Jilin University, Changchun 130041, China

## Abstract

Oxidative stress of the retinal pigment epithelium (RPE) is an essential element contributing to the progression of age-related macular degeneration (AMD). Notably, the activation of Nrf2 is regarded as an effective strategy for controlling oxidation. The novel 2,3-dihydroflavonoid compound farrerol, which is extracted from *Rhododendron*, possesses antioxidant properties. In this study, we investigated the mechanism by which farrerol protects against oxidative damage mediated by hydrogen peroxide (H_2_O_2_) in adult retinal pigment epithelial cell line 19 (ARPE-19) cells. Farrerol supplementation conspicuously reversed H_2_O_2_-related cell damage through declining the generation of intracellular reactive oxygen species (ROS) and MDA and increasing the concentrations of GSH and SOD. According to the results of the apoptosis assay, a farrerol pretreatment decreased the protein expression of the Bax/Bcl-2, cleaved caspase-3, PARP, caspase-8, and caspase-9 proteins. Furthermore, farrerol markedly activated Nrf2, thereby increasing the levels of antioxidant enzymes downstream of Nrf2, such as HO-1, NQO1, and GCLM. Knockdown of Nrf2 with a specific siRNA successfully suppressed farrerol-mediated HO-1 transcription and partially abolished the cytoprotective effect on ARPE-19 cells. Meanwhile, farrerol induced Akt and MAPK phosphorylation in a dose-related way. However, inhibiting Akt and MAPK substantially blocked the cytoprotective functions of farrerol. Therefore, farrerol enhanced Nrf2-mediated cytoprotection of oxidative damage caused by H_2_O_2_, which may be inseparable from the activation of Akt and MAPK.

## 1. Introduction

Age-related macular (AMD) degeneration is an acquired disorder which substantially stimulates the macular area of the retina and causes the patient to gradually lose central vision [1, 2]. Early-stage AMD is generally asymptomatic, although RPE abnormalities, including extracellular drusen deposits, which are located between RPE cells and Bruch's membrane, are clinically observed in the eye's central posterior pole [3]. In addition, late-stage AMD also involves geographic atrophy of RPE or the choroidal neovascular complex. This stage of AMD will make loss of central visual acuity and considerable visual impairment, which may decrease the patient's quality of life [4, 5]. Currently, AMD is still the third main cause of severe irreversible vision loss worldwide [6]. Meanwhile, an estimate of the global prevalence rate also indicated that the number of AMD cases worldwide will reach nearly 300 million by 2040, which constitutes a major public health problem that imposes a substantial burden on society and the economy [7].

Although the specific mechanisms of the development of AMD remain unclear, many studies have shown that oxidative stress and apoptosis acted an essential part in these processes [8]. RPE cells possess strong metabolism and survive in the presence of a large amount of endogenous ROS, consisting of superoxide anions (O2^•-^), hydroxyl radicals (^•^OH), and H_2_O_2_ [9]. Meanwhile, the phagocytosis of photoreceptors and accumulation of lipofuscin may result in further ROS production [10, 11]. In addition, detrimental elements like aging, smoking, and additional UV exposure also increase ROS production. Oxidative damage caused by the long-term accumulation of ROS may lead to RPE cell dysfunction [5]. However, supplementation with antioxidants containing ascorbic acid (vitamin C), provitamin A, and lutein alleviates retinal damage and modulates AMD progression. Therefore, treatments that reduce the oxidative damage to RPE cells are considered an advantageous method to prevent the occurrence and progression of AMD [12, 13]. Nuclear factor erythroid-related factor 2 (Nrf2) is effectively activated to trigger the endogenous antioxidant defense system under stress conditions [14]. Moreover, Nrf2 regulates ROS production and biological metabolism by regulating multiple antioxidants and phase II detoxification [15]. Under oxidative stress conditions, Kelch-like ECH-related protein 1 (Keap1) undergoes modifications that cause a conformational change, thereby restraining the ubiquitination of Nrf2 [9]. Subsequently, Nrf2 translocated to the nucleus and can bind to small tendon fibrosarcoma (sMaf) protein to form a heterodimer. This heterodimer recognizes and binds to ARE, thereby activating the transcription of downstream genes like heme oxygenase-1 (HO-1), NAD(P)H quinone oxidoreductase-1 (NQO1), glutamate-cysteine ligase catalytic subunit (GCLC), and glutamate-cysteine ligase modifier subunit (GCLM) [16, 17]. Additionally, Nrf2 is also regulated via the phosphorylation of Keap1 by several kinases, such as phosphatidylinositol 3 kinase (PI3K)/Akt [18] and mitogen-activated protein kinases (MAPK), including JNK, ERK, and P38 [19, 20]. Therefore, the inhibition of oxidative damage through an approach targeting Nrf2 molecules represents a novel therapeutic strategy for AMD.

Farrerol is a major Nrf2 activator and novel 2,3-dihydroflavonoid compound extracted from *Rhododendron*. As shown in our previous experiments, farrerol possesses biological activities, including antibacterial, anti-inflammatory, and antioxidant functions [21, 22]. According to our previous study, farrerol ameliorates the nephrotoxicity caused by cisplatin accordingly initiating Nrf2 and its downstream, thereby improving oxidative damage, inflammation, and apoptosis [23]. In addition, farrerol also protects against acetaminophen-induced liver damage by regulating Nrf2 and autophagy signaling pathways [24]. Here, we determined the cytoprotection of farrerol on H_2_O_2_-associated oxidation and apoptosis in vitro and further explored the underlying interaction between the Nrf2 regulatory pathway and potential mechanisms.

## 2. Materials and Methods

### 2.1. Reagents and Chemicals

Farrerol (Farr), purity>98%, was obtained from Chengdu Pufei De Biotech Co., Ltd. (Chengdu, China). H_2_O_2_, 2′,7′-dichlorodihydrofluorescein diacetate (DCFH-DA), and Cell Counting Kit-8 (CCK-8) were purchased from Sigma Chemical Company (St. Louis, MO, USA). Antibodies against P-Akt, Akt, P-ERK, ERK, P-P38, P38, JNK, P-JNK, and *β*-actin were purchased from Cell Signaling Technology (Boston, MA, USA). In addition, antibodies against Nrf2, HO-1, NQO1, GCLC, GCLM, PARP, Bax, Bcl-2, caspase-3, caspase-8, and caspase-9 were obtained from Proteintech Group and Abcam (Cambridge, MA, USA). Dulbecco's modified Eagle's medium : Nutrient Mixture F-12 (DMEM/F-12), the antibiotic-antimycotic solution, and trypsin-EDTA were obtained from Invitrogen-Gibco, MBI, and Biofil, respectively. In addition, MDA, GSH, and SOD test kits were obtained from Nanjing Jiancheng Bioengineering Institute (Nanjing, China).

### 2.2. Cell Culture

ARPE-19 cells purchased from the American Type Culture Collection (ATCC, Manassas, Virginia) were maintained in DMEM/F-12 supplemented with 10% fetal bovine serum, 100 U/mL penicillin, and 100 *μ*g/mL streptomycin. Cells were cultured at 37°C in a humidified atmosphere of 5% CO_2_ in air.

### 2.3. CCK-8 Assay

After ARPE-19 cells were grown in 96-well plates (2 × 10^4^ cells/well), various concentrations of H_2_O_2_ (75-1200 *μ*M) or farrerol (5–80 mg/L) were mixed with the media in each well. Furthermore, cells were pretreated with various preselected dosages of farrerol. And then, cells were stimulated with H_2_O_2_ for 24 h. The CCK-8 assay was utilized to verify cell viability.

### 2.4. Measurement of the Malondialdehyde (MDA), Glutathione (GSH), and Superoxide Dismutase(SOD) Levels In Vitro

The cells were preincubated with farrerol at a concentration of 5, 10, or 20 mg/L and coincubated with H_2_O_2_. After 24 hours of incubation, detection kits were used to measure intracellular SOD, GSH, and MDA concentrations according to the instruction of the reagent.

### 2.5. Intracellular ROS Measurement

ARPE-19 cells were grown on 24-well plates (1 × 10^5^ cells/well) for 24 h and then supplemented with or without farrerol for 24 h to assess the ROS scavenging activity of farrerol. The cells were incubated with DCFH-DA (50 *μ*M) and then stimulated with H_2_O_2_ (300 *μ*M). Afterwards, we utilized the excitation wavelength and the emission wavelength to evaluate the fluorescence intensity.

### 2.6. Flow Cytometry

ARPE-19 cells were grown and handled as described previously. Annexin V and propidium iodide (PI) were utilized to quantitate apoptosis according to the manufacturer's instructions. Afterwards, the proportion of apoptotic cells was measured using a flow cytometer.

### 2.7. Western Blot Analysis

ARPE-19 cells were grown on 6-well plates (10 × 10^5^ cells/well) for 24 h, and then, we replaced the original medium with serum-free DMEM/F-12 medium, supplemented with or without farrerol for 24 h to assess the protective effect of farrerol. The cells were collected and lysed according to related reagent instruction. We used the BCA protein assay to measure the protein concentration. After that, proteins were transferred to a PVDF membrane. The membrane was sealed with 5% skim milk. After incubations with the corresponding primary and secondary antibodies, the bands were developed utilizing ECL and quantified using scanning densitometry.

### 2.8. Nrf2 siRNA Transfection

Cells were inoculated into a 6-well plate and grown until the number of cells reached approximately 50% confluence. Cells were then transfected with the control siRNA or Nrf2 siRNA (Santa Cruz, CA, USA) using Lipofectamine 2000 (Thermo Fisher, Madison, USA) according to the manufacturer's instructions. After transfection, the cells were incubated with farrerol for 24 h. The protective effect of farrerol was evaluated using western blot analysis.

### 2.9. Statistical Analysis

Statistical analyses were performed using GraphPad Prism software. All the data described above are reported as means ± standard errors of the means (SEM). Statistical significance was analyzed by one-way analysis of variance (ANOVA) and Bonferroni's test.

## 3. Results

### 3.1. Cytoprotective Effects of Farrerol on H_2_O_2_-Mediated Damage in ARPE-19 Cells

**Figure 1:**
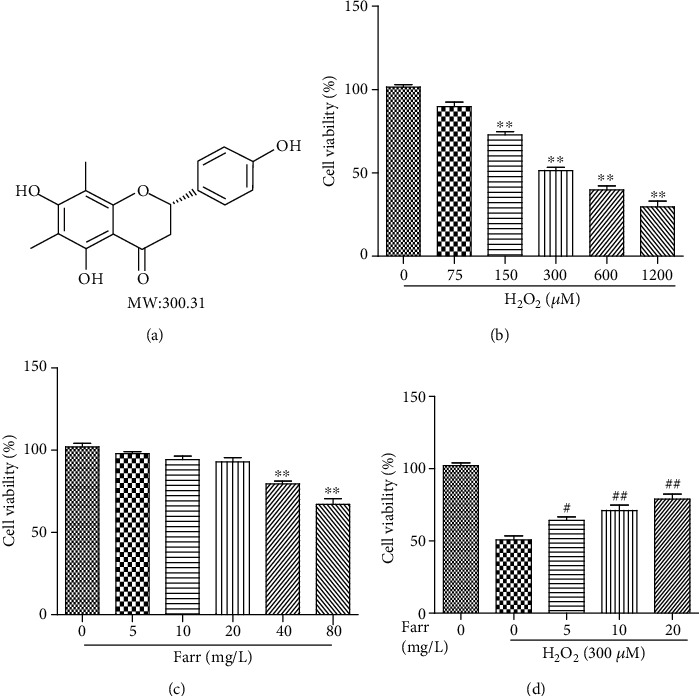
Impacts of H_2_O_2_ and farrerol on ARPE-19 cells' viability. (a) Chemical structure of farrerol (Farr) (MW = molecular weight). ARPE-19 cells were dealt with different doses of H_2_O_2_ (75-1200 *μ*M) (b) or farrerol (5–80 mg/L) (c) for 24 h. And then, the CCK-8 was utilized to calculate the cell viability. (d) Cells were pretreated with farrerol at concentrations of 5, 10, and 20 mg/L for 1 h followed by 300 *μ*M H_2_O_2_ stimulation for 24 h, and then, CCK-8 was utilized to evaluate cell survival. Similar results were illustrated from three independent experiments. ^∗^*p* < 0.05 and ^∗∗^*p* < 0.01 compared with the control group; ^#^*p* < 0.05 and ^##^*p* < 0.01 compared with the H_2_O_2_ group.

Using H_2_O_2_ to imitate cytoprotection influence of RPE cells on oxidation is a well-known and ideal model [9]. Thus, the CCK-8 was employed to detect the cytotoxicity and cytoprotective effects of H_2_O_2_ and farrerol, respectively. First and foremost, we investigated the cells' viability in different doses of H_2_O_2_. Cell viability was considerably reduced in response to 300 *μ*M H_2_O_2_ compared with the control group (Figure 1(b)). Hence, we utilized H_2_O_2_ (at a dose of 300 *μ*M) in subsequent experiments to appraise the cytoprotection of farrerol against H_2_O_2_-mediated damage. Furthermore, as shown in Figure 1(c), a remarkable difference in viability was not observed in cells pretreated with farrerol at concentrations ranging from 5 to 10 mg/L; however, at a concentration of 20 mg/L, this may result in statistical difference of cell viability. Based on statistical analyses described above, the cells were pretreated with farrerol (0–20 mg/L) and then incubated with 300 *μ*M H_2_O_2_. The farrerol supplement, particularly at a dose of 20 mg/L, attenuated the cytotoxicity of H_2_O_2_ (Figure 1(d)).

### 3.2. Farrerol Mitigates the H_2_O_2_-Induced Oxidation of ARPE-19 Cells

**Figure 2:**
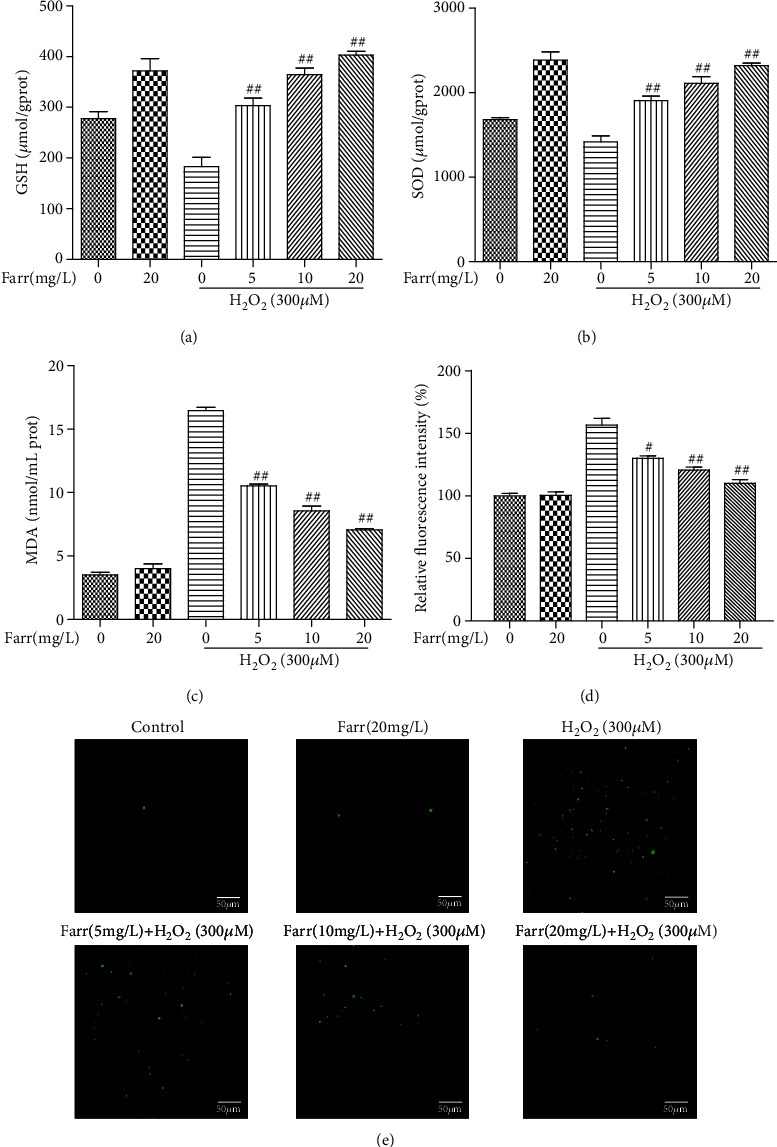
Effects of farrerol on H_2_O_2_-mediated oxidation in vitro. ARPE-19 cells were cultured and collected to measure the levels of GSH (a), SOD (b), and MDA (c). After staining ARPE-19 cells with the ROS fluorescent probe, they were stimulated with H_2_O_2_. Subsequently, a fluorescence microscope was using to observe the fluorescence (d). ROS-positive cells were quantified and analyzed (e). Similar results were obtained from three independent experiments. ^#^*p* < 0.05 and ^##^*p* < 0.01 compared with the H_2_O_2_ group.

For many years, oxidative damage was regarded as the main mechanism underlying AMD pathophysiology in the RPE [25]. H_2_O_2_ increased MDA levels and decreased SOD and GSH levels. In contrast, farrerol alleviated H_2_O_2_-mediated oxidative damage by reducing the MDA concentrations and enhancing the SOD and GSH levels compared to the H_2_O_2_ treatment alone (Figures 2(a)–2(c)). In addition, farrerol distinctly decreased intracellular ROS levels and cell death, as evidenced by the results of the DCFH-DA staining (Figures 2(d) and 2(e)).

### 3.3. Farrerol Attenuates H_2_O_2_-Induced Apoptosis in ARPE-19 Cells

**Figure 3:**
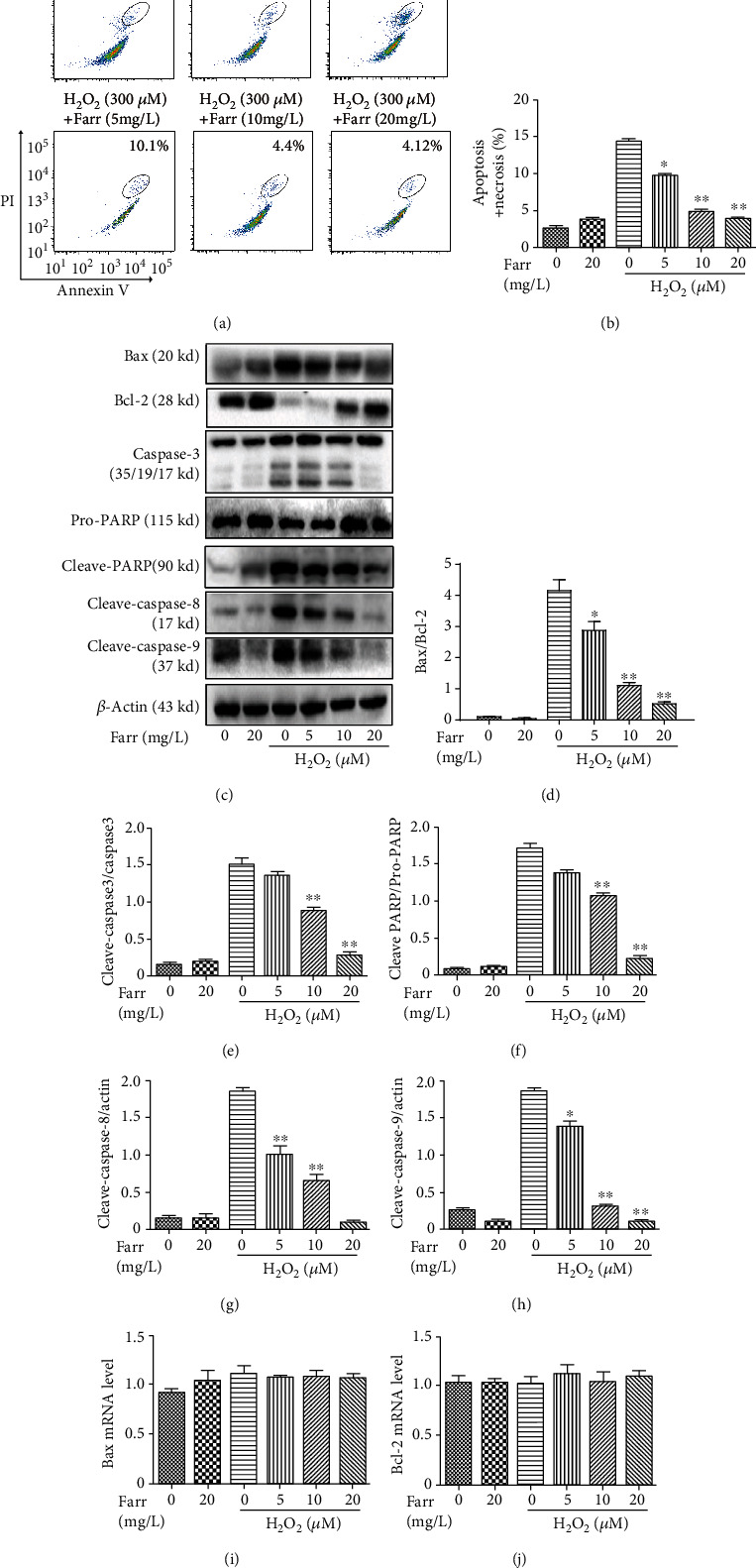
Inhibitory impact of farrerol on H_2_O_2_-induced apoptosis in vitro. (a, b) Quantitation of apoptotic cells with flow cytometry. (c) Levels of the Bax/Bcl-2 (d), caspase-3 (e), cleaved PARP/pro-PARP (f), cleaved caspase-8 (g), and cleaved caspase-9 (h) proteins were assessed utilizing western blotting. The mRNA expression of Bax (i) and Bcl-2 (j) in ARPE cells treated with H_2_O_2_ (300 *μ*M) and different concentrations of farrerol (5, 10, and 20 mg/L). All of the data represent the means of three independent experiments. ^∗^*p* < 0.05 and ^∗∗^*p* < 0.01 compared with the H_2_O_2_ group. *β*-Actin was used as an internal control.

For a more comprehensive analysis of the potential cytoprotection mechanism of farrerol on AMD, we investigated the apoptosis of ARPE-19 cells treated with H_2_O_2_ using flow cytometry. As shown in Figures 3(a) and 3(b), H_2_O_2_-mediated apoptosis was alleviated by the farrerol pretreatment in vitro. Moreover, western blot was conducted to evaluate the levels of apoptosis-associated proteins. As shown in Figures 3(c) and 3(d), it was found that pretreatment with farrerol can increase the antiapoptotic protein Bcl-2, while significantly reducing the levels of proapoptotic proteins Bax, caspase-3, and cleaved PARP. Notably, 5 mg/L of farrerol cannot significantly reduce the content of cleaved PARP and caspase-3. In addition, western blot analysis also showed that farrerol decreases the levels of cleaved caspase-8 and cleaved caspase-9 in ARPE-19 cells induced by H_2_O_2_ (Figures 3(g) and 3(h)). However, qPCR analysis showed that the mRNA expression levels of apoptosis-related proteins Bax and Bcl2 did not change significantly in ARPE-19 cells (Figures 3(i) and 3(j)).

### 3.4. Farrerol Activates the Nrf2 Regulatory Pathway In Vitro

**Figure 4:**
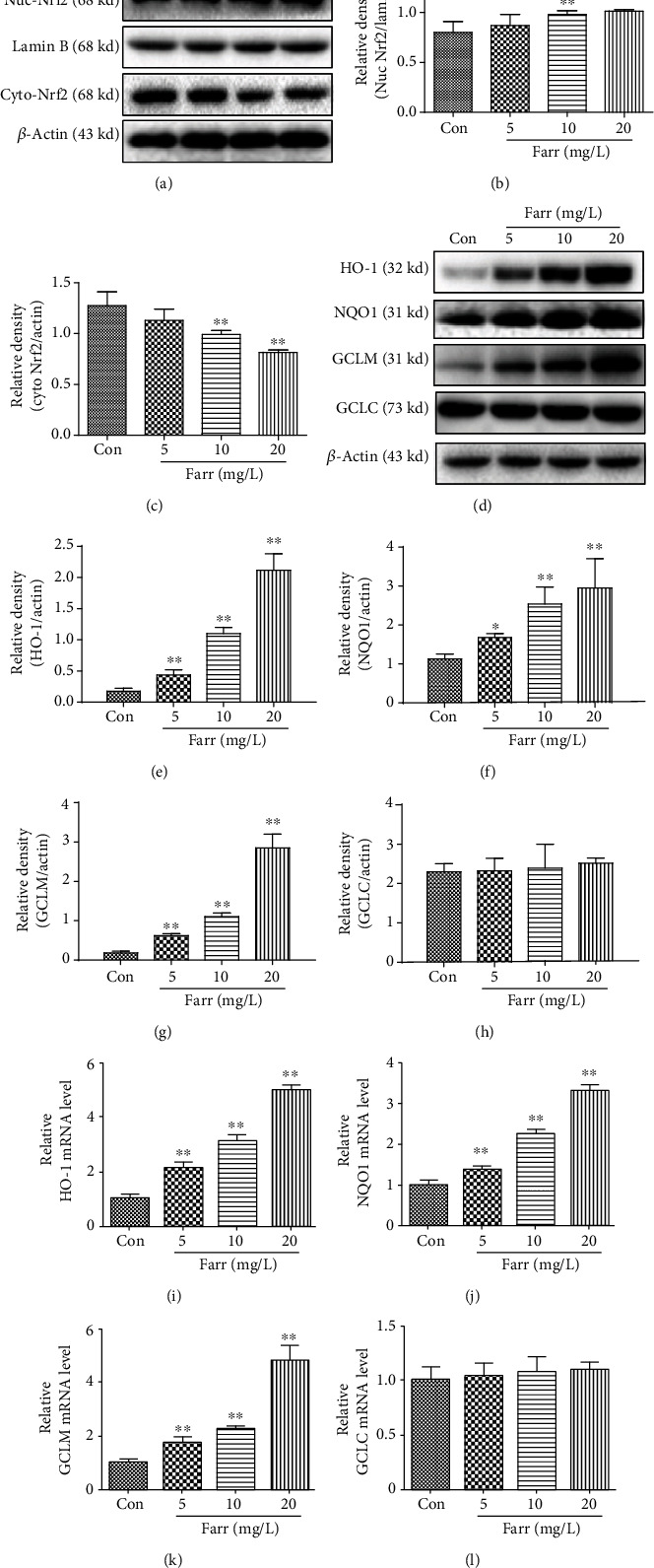
Activation of the Nrf2 signaling pathway is involved in the cytoprotective influence of farrerol on H_2_O_2_-related cell damage. (a) Cells were coincubated with different doses of farrerol for 24 h, and the nuclear (b) and cytoplasmic (c) levels of Nrf2 were then determined using western blotting. (d) Representative western blots showing levels of the HO-1 (e), NQO1 (f), GCLM (g), and GCLC (h) proteins are shown. The mRNA expression of HO-1 (i), NQO1 (j), GCLM (k), and GCLC (l) in ARPE cells treated with different concentrations of farrerol (5, 10, and 20 mg/L). The results represent the average values of three independent experiments. ^∗^*p* < 0.05 and ^∗∗^*p* < 0.01 compared with the control group. *β*-Actin served as an internal control.

We also evaluated the antioxidant properties of farrerol by determining the localization of Nrf2 and the generation of the downstream molecules HO-1, NQO1, GCLC, and GCLM in vitro. First, we studied the translocation of Nrf2 in cells treated with different concentrations (5, 10, and 20 mg/L) of farrerol for 24 h. The translocation of Nrf2 was significantly increased, and the most remarkable change was observed in cells treated with 20 mg/L farrerol (Figures 4(a)–4(c)). Meanwhile, as shown in Figures 4(d)–4(g) and 4(i)–4(k), farrerol also increased the mRNA and protein generation of HO-1, NQO1, and GCLM in cells in a dose-dependent way. However, in our experiments, we did not find significant changes in levels of GCLC (Figures 4(h) and 4(l)). Based on these results, farrerol increased the expression of Nrf2 and its target genes, HO-1, NQO1, and GCLM, in a concentration-related way.

### 3.5. The Protection of Farrerol from H_2_O_2_-Induced Apoptosis Depends on the Nrf2 Signaling Pathway in ARPE-19 Cells

**Figure 5:**
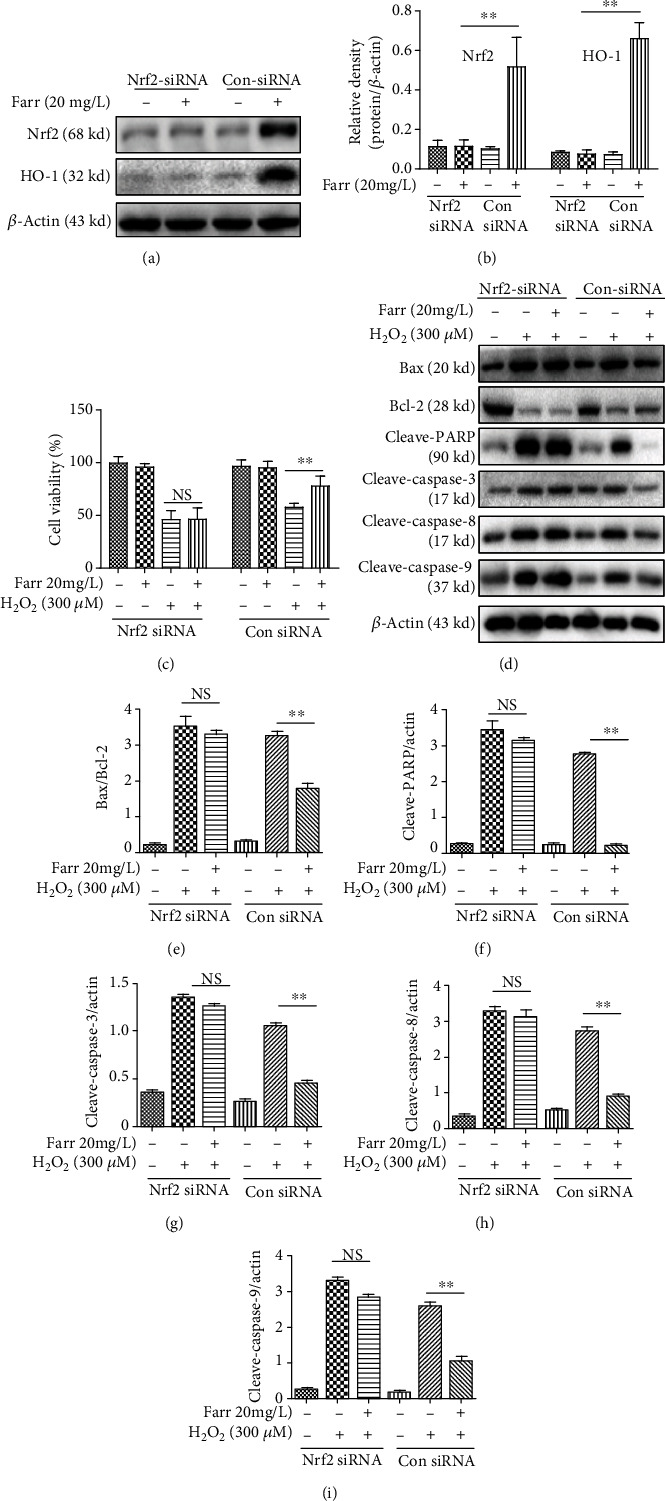
Nrf2 knockdown weakens the protective effect of farrerol in vitro. Cells were transfected with an Nrf2 siRNA or Nrf2-negative control siRNA for 1 h and exposed to farrerol for 24 h, and the protein levels were subsequently detected with western blotting. (a, b) The levels of Nrf2 and HO-1 were determined. (c) Analysis of the cytoprotective effect of farrerol using the CCK-8 assay. Immunoblotting (d) and quantification of Bax/Bcl-2 (e), cleaved PARP (f), cleaved caspase-3 (g), cleaved caspase-8 (h), and cleaved caspase-9 (i) protein expression following transfection with siNrf2. All results are presented as means ± SEM (*n* = 3). ^∗^*p* < 0.05 and ^∗∗^*p* < 0.01 compared with the H_2_O_2_ group. NS = not specific. *β*-Actin served as an internal control.

We used a siRNA targeting Nrf2 to silence the expression of Nrf2 in vitro and to further investigate the function of Nrf2 in RPE cells. Farrerol reversed the H_2_O_2_-induced decrease in the levels of the HO-1 protein, but the siNrf2 treatment can abolish this protective effect of farrerol (Figures 5(a) and 5(b)). Moreover, the downregulation of Nrf2 significantly decreased the protective effect of farrerol in vitro (Figure 5(c)). In addition, the effects of farrerol on the protein expression related to apoptosis and antiapoptosis were not observed when Nrf2 expression was suppressed using siNrf2 when compared with the siNrf2 alone group (Figures 5(d)–5(i)). These indicated that farrerol's protection against H_2_O_2_-induced cell damage was dependent on the expression of Nrf2.

### 3.6. Akt and MAPK Activation Is Involved in the Protective Effect of Farrerol

**Figure 6:**
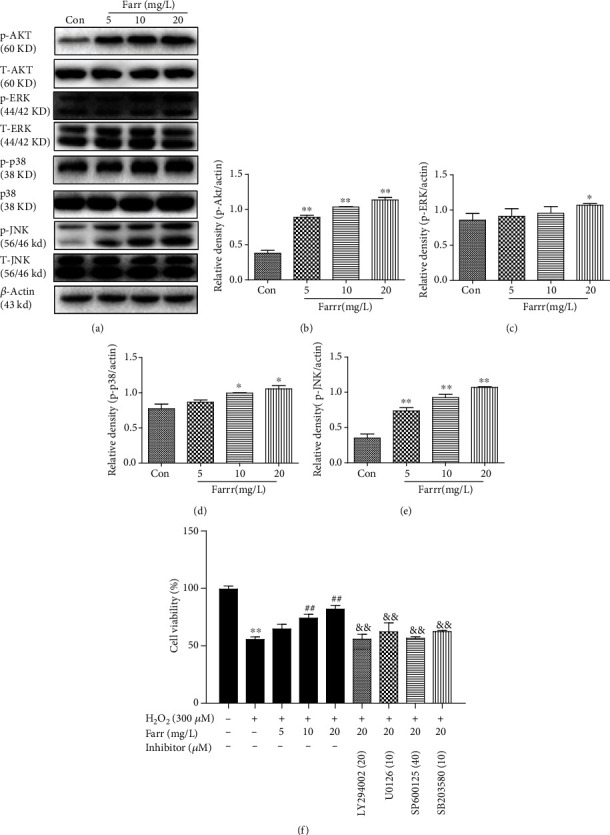
Akt and MAPK signaling pathways are related to the protective impacts of farrerol in vitro. (a–e) Quantification of the relative levels of the P-Akt/T-Akt, P-ERK/T-ERK, P-P38/T-P38, and P-JNK/T-JNK proteins using western blotting. (f) Protective effect of farrerol on cells was investigated using the CCK-8. Cells were treated with farrerol and then incubated with or without LY294002 (20 *μ*M), UO126 (10 *μ*M), SP600125 (40 *μ*M), and SB203580 (10 *μ*M) for 4 h. All experiments were repeated three times. ^∗^*p* < 0.05 and ^∗∗^*p* < 0.01 compared with the control group; ^#^*p* < 0.05 and ^##^*p* < 0.01 compared with the H_2_O_2_ group. ^&^*p* < 0.05 and ^&&^*p* < 0.01 compared with the H_2_O_2_ (300 *μ*M)+farrerol (20 mg/L) group. *β*-Actin served as an internal control.

We investigated whether and how the Akt and MAPK signaling participates in the cytoprotective effect of farrerol on cells. As shown in Figures 6(a)–6(e), western blots were performed to observe Akt and MAPK activation in farrerol-treated cells. When compared with the control group, farrerol supplement piled up the phosphorylation of Akt and MAPK in ARPE-19 cells in a concentration-dependent way. In addition, an Akt inhibitor (LY294002), JNK inhibitor (SP600125), ERK inhibitor (UO126), and P38 inhibitor (SB203580) nearly completely abolished the farrerol cytoprotection on ARPE-19 cells dealt with H_2_O_2_ (Figure 6(f)). Thus, Akt and MAPK activation is critical for farrerol-induced Nrf2 nuclear translocation and cytoprotection.

## 4. Discussion

As a retinal disorder, AMD mainly causes irreversible blindness among the aged population in the developed world [26]. Approximately 11 million of Americans suffer from AMD, and this figure may continuously increase and will probably become a global medical burden [27]. A feasible therapy for AMD is not available, and thus, the demand for new treatments has become increasingly urgent. Notably, AMD is a complex disease caused by genetic and environmental factors [28]. Although the precise mechanism of its pathogenesis is unknown, the progressive degeneration of the macular RPE cells in the retina may cause AMD. The degeneration of the RPE involves crosstalk between oxidation and apoptosis pathways and is a well-known essential factor contributing to the pathogenesis of AMD [29].

The retina is a tissue with a high oxygen consumption rate. Its photoreceptor cells are continuously exposed to oxygen and light, and thus, they are more vulnerable to oxidative stress [30]. Excessive ROS production induced by chronic oxidative damage is the main factor leading to AMD, and its pathophysiology may cause oxidative damage to cellular components and severely destroy a proportion of RPE cells. Consistent with these findings, H_2_O_2_ significantly increased ROS production in the present study (Figures 2(d) and 2(e)). Moreover, antioxidants also remarkably decrease the rate of AMD progression in the clinic [29, 31]. Thus, a new method for inhibiting oxidative stress would be a potential treatment for AMD. The production of MDA and the consumption of GSH and SOD have been frequently used as indicators of oxidative damage. In our present study, H_2_O_2_-induced oxidation resulted in higher levels of MDA and lower levels of SOD and GSH (Figures 2(a)–2(c)).

Farrerol, a new 2,3-dihydroflavonoid compound extracted from *Rhododendron*, possesses antibacterial, anti-inflammatory, antioxidant, and other biological activities [21, 22]. As shown in Figures 2(a)–2(c), farrerol effectively reversed the changes in the indicators described above. In addition, farrerol significantly reduced the H_2_O_2_-induced increase in ROS levels in cells (Figures 2(d) and 2(e)). Consistent with these findings, farrerol visibly attenuated oxidative damage and potentially represents a treatment for H_2_O_2_-induced cytotoxicity (Figure 1(d)). In addition, a large amount of accumulated ROS may cause mitochondrial dysfunction in RPE cells and induce apoptosis [32]. In our study, farrerol significantly decreased apoptosis compared with the H_2_O_2_ treatment alone, as determined using flow cytometry (Figures 3(a) and 3(b)). In addition, we also observed the levels of the Bax/Bcl-2, cleaved caspase-3, and cleaved PARP protein by performing western blot analyses. The levels of these apoptosis-related proteins were distinctly increased in the H_2_O_2_ treatment group, and the farrerol pretreatment substantially reduced their levels (Figures 3(c)–3(f)). As we all know, caspase-3, as an important effector molecule in the apoptosis pathway, can trigger mitochondrial and the death ligand pathways by interacting with caspase-9 and caspase-8, respectively [33]. In the mitochondrial activation pathway, mitochondrial cytochrome c can be released into the cytoplasm and cause the cleaved caspase-9 to activate the expression of downstream caspase-3 [34]. In the death ligand activation pathway, death receptors (such as FasL and FasR) can cause apoptosis to activate downstream cleaved caspase-8, thereby activating the expression of caspase-3 [35]. In the following study, western blot analysis showed that farrerol downregulated the levels of cleaved caspase-3, cleaved caspase-8, and cleaved caspase-9 in ARPE-19 cells induced by H_2_O_2_. However, the protective effects of farrerol on cleaved caspase-8 and cleaved caspase-9 were not observed when Nrf2 expression was suppressed using siNrf2 when compared with the siNrf2 alone group (Figures 5(d)–5(i)). These results indicated that farrerol improves H_2_O_2_-induced ARPE-19 cell damage by restraining death receptors and mitochondrial apoptotic pathways.

As shown in our previous study, farrerol ameliorates renal toxicity caused by cisplatin and acetaminophen-induced liver damage by activating the Nrf2 signaling to improve oxidative damage [23, 24]. With the aim of further studying the pharmacological effects of farrerol and based on the aforementioned results, we investigated antioxidant molecules to explore the mechanism underlying the interaction between Nrf2 and antioxidants. The Nrf2 signaling pathway is required to regulate the expression of antioxidant and antiapoptosis-related enzymes, and this pathway plays a considerable role in maintaining antioxidant homeostasis [36, 37]. Under stress conditions, the newly synthesized Nrf2 translocates to the nucleus and subsequently activates downstream antioxidant genes to inhibit ROS production [38]. As shown in the present study, farrerol protected cells from H_2_O_2_-mediated oxidative stress by inducing the nuclear translocation of Nrf2 (Figures 4(a)–4(c)) and increasing its downstream like HO-1, NQO-1, and GCLM (Figures 4(d)–4(g) and 4(i)–4(k)). Additionally, the silencing of Nrf2 partially abolished the cytoprotective effects of farrerol (Figure 5(c)) and decreased the HO-1 level (Figures 5(a) and 5(b)). A potential explanation for this finding is that farrerol-associated cytoprotective activities are carried out through the Nrf2/HO-1 pathway to some extent. Notably, the expression of ARE-dependent genes is also induced by activated kinase pathways (such as MAPK and PI3K/Akt) in cells [18–20]. In our present study, the farrerol treatment effectively induced the necessary phosphorylation of Akt and MAPK (Figures 6(a)–6(e)), which is crucial for subsequent Nrf2 activation in RPE cells. However, the Akt inhibitor (LY294002), JNK inhibitor (SP600125), ERK inhibitor (UO126), and P38 inhibitor (SB203580) nearly completely abolished the cytoprotective impact of farrerol in vitro stimulated with H_2_O_2_ (Figure 6(f)). Based on these results, Akt and MAPK activation is related to the cytoprotective effect of farrerol on RPE cells subjected to H_2_O_2_-induced oxidative damage and subsequent apoptosis.

Taken together, the results of this study indicated that farrerol has novel functions that protect RPE cells from H_2_O_2_-associated oxidation and apoptosis by inhibiting ROS generation. Farrerol ameliorates H_2_O_2_-induced cell death by increasing Nrf2/HO-1 generation via activating Akt and MAPK in ARPE-19 cells. Thus, farrerol shows promise in the treatment or prevention of AMD.

## Data Availability

All datasets analyzed for this study are included in the article material.
